# Characteristics of adolescents living with HIV receiving care and treatment services in antiretroviral therapy clinics in Cambodia: descriptive findings from a cross-sectional study

**DOI:** 10.1186/s12913-018-3580-1

**Published:** 2018-10-16

**Authors:** Siyan Yi, Sovannary Tuot, Khuondyla Pal, Vohith Khol, Say Sok, Pheak Chhoun, Laura Ferguson, Gitau Mburu

**Affiliations:** 1 Saw Swee Hock School of Public Health, National University of Singapore and National University Health System, Singapore, Singapore; 2 KHANA Center for Population Health Research, No. 33, Street, Phnom Penh, 71 Cambodia; 3 Center for Global Health Research, Touro University California, Vallejo, CA USA; 4 National Center for HIV/AIDS, Dermatology and STD, Phnom Penh, Cambodia; 5 Department of Media and Communication, Royal University of Phnom Penh, Phnom Penh, Cambodia; 6 Institute for Global Health, Keck School of Medicine, University of Southern California, Los Angeles, CA USA; 7 Division of Health Research, Lancaster University, Lancaster, UK

**Keywords:** Adolescents, HIV, Healthcare transition, Pediatric care, Adult services, Cambodia

## Abstract

**Background:**

Adolescents living with HIV experience worse HIV care outcomes compared to adults, especially during transition from pediatric to adult care. However, data regarding adolescents are limited. This paper describes and compares characteristics of male and female adolescents living with HIV preparing for transition from pediatric to adult care in Cambodia.

**Methods:**

This cross-sectional study was conducted in August 2016 among 328 adolescents aged 15–17, randomly selected from 11 antiretroviral therapy (ART) clinics. Data were collected using a structured questionnaire, and descriptive analyses were conducted to compare characteristics of male and female adolescents.

**Results:**

Of total, 55.2% were male, and 40.8% were living with parents. Majority (82.6%) got HIV infection from their mothers. Overall, adolescents had received ART for an average of 8.4 years, and HIV care for 9.5 years. Additionally, 82.4% were on first line ART regimen. Mean CD4 count from the most recent test was 672 cells/mm^3^, and viral load was 7686 copies/mL. Overall, 95.6% were adherent to ART on Visual Analogue Scale. About half (50.7%) had never disclosed their HIV status to anyone, while the remaining had disclosed it to their siblings (24.2%), friends (13.0%), schoolteachers (2.4%), or other (5.8%). A fifth reported having had boy or girlfriends, but few (2.1%) had ever had sexual intercourse. Females were more likely to have been engaged in sexual intercourse, and none reported having used a condom in their last intercourse. Few participants reported having ever used tobacco (1.8%), or any kind of illicit drugs (0.9%), but almost a fifth (20.7%) had a history of alcohol use. The majority (82.1%) were aware that they were receiving ART. HIV-related knowledge was suboptimal among the sample.

**Conclusions:**

This study provides a snapshot of immunological, virological, adherence, and disclosure outcomes that should be tracked during and following healthcare transition to evaluate the effectiveness of the transition program. Findings showed high ART adherence, low likelihood of disclosure outside of family circles, sub-optimal condom use, and poor knowledge of HIV. To provide individualized support for healthcare transition, pediatric and adult clinics need to ensure that these characteristics are taken into account.

## Background

Over 2.1 million adolescents (aged 10–19 years) are living with HIV globally [1], and this number continues to grow, mainly due to ongoing mother-to-child transmission, increased survival as a result of antiretroviral therapy (ART), and sexual transmission among adolescents [2]. In Cambodia, where HIV prevalence was 0.28% among the general population aged 15–49 years in 2014 [3], there were an estimated 8512 children and adolescents living with HIV at the end of 2013 [4].

Although there have been remarkable achievements in the global HIV response, the progress in HIV prevention, care, and treatment among adolescents aged 10–19 years is lagging behind other age groups [5, 6]. While mortality among people living with HIV in all other age groups is decreasing, that of adolescents living with HIV is increasing [6]. Reasons for poor HIV outcomes in adolescents have included poor long-term immunologic recovery [7], poor adherence to ART due to treatment fatigue and marginalization [8], delayed diagnosis [9], stigma [10, 11], difficulties with disclosure [12, 13], lack of awareness of serostatus [14], denial of care by legal parents or guardians for a myriad of personal reasons such as fear of the child’s or parents’ HIV status disclosure and/or structural factors such as poverty or alternative beliefs in treatment [15], and restrictive legal and policy environments that impede their own ability to consent to services [16].

Of particular concern is the unique issue of transition from pediatric to adult clinics. The circumstances and characteristics of adolescents during transition is increasingly being recognized as a significant determinant of their HIV care outcomes [17]. For instance, transition between pediatric to adult services is often accompanied by significant loss to follow-up [18]. During this time, adolescents are gaining mental, physical maturity, independence [19, 20], and seeking autonomy and independence from their family and attachment to their peer groups [21]. These circumstances may intersect with substance use, self-determination, burgeoning responsibility, mental health issues, psychosocial delays, sexual behaviors, and childbearing to affect their adherence to ART, viral suppression, and eventual ability to transition successfully into adult care [20–24]. The magnitude of the HIV epidemic among adolescents and their relatively poor clinical outcomes, especially during transition, are projected to worsen unless adequate steps are taken and appropriate transition models are considered to address this population’s unique needs [2, 21, 25].

Healthcare transition can be defined as ‘the purposeful, planned movement of adolescents and young adults with chronic medical conditions from a child-centered to an adult-oriented health care system’ [21, 23, 24]. According to the World Health Organization (WHO), healthcare transition preparedness is determined by adolescents’ ability to demonstrate basic healthcare awareness, awareness of their status, understanding their disease, and development of own health management skills [26]. However, number of research regarding adolescents’ healthcare transition in the context of HIV, especially in low- and middle-income countries is relatively limited [6, 24]. This is particularly concerning given that adolescents living with HIV in low- and middle-income countries are disproportionately affected by disparities in HIV mortality [27]. Scarcity of data around transition is further exacerbated by current demographic disaggregation of adolescents into 10–14 and 15–24 [1, 6], which limits the availability of data regarding the needs, conditions, and situations of under-18 year olds living with HIV, especially in resource-limited settings [21, 28, 29]. Without adequate data, understanding the circumstances of adolescents living with HIV and tailoring services, which are known to improve treatment outcomes [30], will be challenging. Generating data around adolescents’ transition readiness is therefore critical to informing appropriate policies and services.

In Cambodia, transition of adolescents from pediatric into adult care is supposed to occur at the age of 15 years. However, in practice, a large proportion of adolescents living with HIV aged 15–17 remain in pediatric clinics. Exploratory studies among adolescents in healthcare transition have only started emerging in the last three years. In these studies, stigma and a lack of confidentiality were the main challenges for adolescents during the transition [31, 32]. However, these studies were of limited scope with small sample sizes, limiting their potential to inform service delivery and policy interventions [33]. The extent to which transition experiences are affected by the differential sexual debut and maturation across gender [6] is unknown, yet this information would be important to tailor healthcare transition. In response to this gap, a larger study was designed to explore adolescents’ preparedness for the transition from pediatric to adult HIV care settings in Cambodia. This paper aimed to describe the characteristics and level of preparedness for the transition and examine gender differences among this young population.

## Methods

### Study design

This study was conducted in 2016 and consisted of a cross-sectional survey, complemented with data from routine clinical data retrieved from medical records in the selected ART clinics. The details of the larger study have been published elsewhere [34, 35].

#### Study population

This study included adolescents living with HIV, who were accessing services from 11 pediatric ART clinics and being prepared for transition to adult HIV care. Adolescents were included in the study if they were: (1) aged between 15 and 17 years; (2) receiving HIV treatment and care services from the selected ART clinics; (3) able to communicate in Khmer; (4) allowed by a consenting parent or guardian to participate; (5) able to present themselves on the day of the interview; and (6) sufficiently physically and mentally stable to participate in the study.

#### Study sites

Study sites were selected from a universe of 39 ART clinics that provided treatment and care to adolescents living with HIV nationally. Of these, ART sites that provided treatment and care to fewer than 10 adolescents (*n* = 24) were excluded to reduce data collection cost. The remaining 11 ART clinics that were providing care and treatment to 598 adolescents living with HIV in the capital city and 10 provinces were included in the study. These diverse sites were scattered throughout the country and were located in rural, urban, and peri-urban areas.

#### Sample size

The sample size was calculated using a cluster-sampling method [36] and was based primarily on an assumption that 60% of the sampled adolescents would possess a high level of preparedness to transition to adult care. Assuming a design effect of 1.4 and a 10% dropout or refusal rate, a minimum sample size of 320 was required for the study with a confidence interval of 4.3%.

#### Sampling procedure

A two-stage 30 × 10 cluster sampling method was used [37]. Because the size of the ART clinics varies, clusters were selected using probability proportional to size sampling method [36], which ensured that adolescents in larger sites had the same probability of being included in the sample as those in smaller sites. A list of all ART sites in the country was compiled from which those with more than 10 adolescents was used to select the clusters. The adolescent population size of each ART clinic and sampling interval was then determined, and a random number was chosen as a starting point on the list to begin cluster selection.

#### Participant recruitment

Local coordinators worked closely with the database officer of each ART clinic to randomly select the eligible participants from the database using a table of random numbers. The list of selected adolescents was compiled. These adolescents were allocated a unique identification number, which was based around their clinic ID. As part of initial screening process, the local study coordinators contacted adolescents through their parent or guardian to inform them about the research objectives. Those who accepted the invitation to participate were provided with an appointment with details regarding the place, date, and time of the interview. Twelve selected adolescents refused the participation due to their school commitment and were replaced by the next adolescents on the name list.

#### Variables and measurements

The study collected a variety of data and measures informed by existing literature [23, 38]. A structured questionnaire was administered to gather data related to demographic characteristics, ART clinic category (pediatric or adult), ART and immunological and virological statuses, adherence to ART, level of preparedness to be transferred to an adult clinic, healthcare coverage and access, HIV knowledge, substance use, sexual behaviors, access to health services, disclosure, among others.

To measure adherence to ART, we used Visual Analogue Scale [39]. The participant was asked to think back over the past three days and identify the times when he or she either missed a dose or took it at the wrong time. The interviewer showed the participant a copy of the scale. While placing their finger on the appropriate number, the interviewer told the participant that, if he or she had taken all medicine doses, to point to 10. If the participant missed all the medicine, he or she would point to 0—if the scale is marked off at 4, then the percentage adherence would be 40%.

The questionnaire was pretested and validated for contents and language with 20 adolescents at one ART site that was not part of the study, and subsequently revised based on their feedback.

#### Data collection

Data were collected by two teams of interviewers and moderators, who were experienced in data collection, and who had been trained on the study protocol, interview skills, and research ethics including specific issues related to research with human participants [40] and young people [41]. These teams were monitored by field supervisors who reviewed questionnaires at the end of each day to ensure entries were appropriately completed. Questionnaires with incomplete or queried entries were re-validated with participants within 24 h. In addition, we collected immunological and clinical data, including ART history and laboratory values for CD4 count and viral load from medical records.

#### Data analyses

Data were double-entered by data clerks as continuous or as coded categorical variables into Epi Data version 3 (Odense, Denmark). Frequency distribution of responses was determined for ordinal and categorical variables, and a mean value with a standard deviation (SD) was calculated for continuous variables. Descriptive analyses were conducted to determine the proportion of adolescents with each variable. Chi-square test, Fisher’s exact test, or Student’s *t*-test, characterized based on the nature of variables being analysed, were used to compare relevant variables between male and female adolescents. *P*-values of less than 0.05 were considered to be statistically significant. SPSS version 22 (IBM Corporation, New York, USA) was used for all statistical analyses.

#### Ethical consideration

This study was conducted in keeping with guidelines related to research involving human subjects [40]. All researchers and data collectors were re-trained on the ethical issues prior to data collection. At the eligibility screening stage, verbal assent from adolescents and verbal consent from a parent or guardian was obtained. Participants were interviewed without their parent/guardian in a private room. In case of need, his/her parent/guardian was called for clarification. Confidentiality was strictly protected by removing all personal identifiers from the questionnaires and assigning each participant a unique code. All participants were provided with a token of 5 USD for transportation and time compensation. The study protocol and tools were approved by the National Ethics Committee for Health Research, Ministry of Health in Cambodia (Ref: 297NECHR).

## Results

### Characteristics of the study sample

Table 1 shows socio-demographic characteristics of the study sample. The study included 328 adolescents living with HIV with a mean age of 15.8 (SD = 0.8) years. Male adolescents comprised 55.2% of the sample. About half (50.7%) reported that they had completed secondary school. Participants were currently living with parents (40.8%), relatives (33.6%), grandparents (15.7%), in an orphanage (8.1%), or with Buddhist monks, neighbours, or foster parents (1.8%). Less than half (47.5%) reported that their mother was still alive, and 42.6% reported that their father was still alive. The formal education level of parents was generally low, with only 19.4% of mothers and 20.9% of fathers having completed high school or university education. The participants reported that their main caregivers were their biological parents (55.6%), relatives (32.2%), siblings (6.7%), orphanage/NGO staff (3.3%), or grandparents (2.2%). More than half of them (52.5%) did not know the education levels of their caregivers other than their parents, while 21.6% reported that their main caregiver had completed high school or university education. Besides the father’s education level (*p* = 0.04) and main caregiver’s education level (*p* ≤ 0.001), there was no statistically significant difference between male and female participants in other variables.

**Table 1:** Characteristics of adolescents living with HIV from 11 ART clinics in Cambodia

Socio-demographic characteristics	Total (*n* = 328)	Males (*n* = 169)	Females (*n* = 159)	
	*n* (%)	*n* (%)	*n* (%)	*P*-value^*^
Mean age (in years)	15.9 ± 0.8	15.8 ± 0.8	16.0 ± 0.8	0.06
Level of formal education				0.76
Primary school or lower	92 (28.0)	50 (20.7)	42 (26.4)	
Secondary school	168 (51.3)	86 (50.9)	82 (51.6)	
High school or higher	68 (20.7)	33 (19.5)	35 (22.0)	
Currently living with:				0.33
Parents	140 (42.7)	73 (43.2)	67 (42.1)	
Grandparents	49 (14.9)	29 (12.7)	20 (12.6)	
Relatives	106 (32.3)	54 (32.0)	52 (32.7)	
In an orphanage	27 (8.2)	12 (7.1)	15 (9.4)	
Other	6 (1.8)	1 (0.6)	5 (3.1)	
Mother is still alive				0.18
No	167 (60.9)	80 (47.3)	87 (54.7)	
Yes	161 (49.1)	89 (52.7)	72 (45.3)	
Mother’s education level				0.12
No education	20 (12.7)	7 (8.1)	13 (18.1)	
Primary school	53 (33.5)	27 (31.4)	26 (36.1)	
Secondary school	24 (15.2)	14 (16.3)	10 (13.9)	
High school or higher	24 (15.2)	12 (14.0)	12 (16.7)	
Don’t know	37 (23.4)	26 (30.2)	11 (15.3)	
Father is still alive				0.82
No	198 (60.4)	101 (59.8)	97 (61.0)	
Yes	130 (39.6)	68 (40.2)	62 (39.0)	
Father’s education level				0.04
No education	9 (5.6)	4 (6.2)	3 (4.3)	
Primary school	33 (26.2)	12 (18.8)	21 (33.9)	
Secondary school	18 (14.3)	8 (12.5)	10 (16.1)	
High school or higher	28 (24.3)	9 (14.1)	14 (22.6)	
Don’t know	45 (35.7)	31 (48.4)	14 (22.6)	
Main daily caregiver:				0.57
Parent	187 (57.0)	102 (60.4)	85 (53.5)	
Grand parent	5 (1.5)	2 (1.2)	3 (1.9)	
Sibling	24 (7.3)	9 (5.3)	15 (9.4)	
Relatives	100 (30.5)	50 (29.6)	50 (31.4)	
Orphanage/NGO staff	12 (3.7)	6 (3.6)	6 (3.8)	
Main caregiver’s education level:				< 0.001
Primary school or lower	14 (7.2)	2 (2.1)	12 (12.1)	
Secondary school	40 (20.5)	8 (8.3)	32 (32.3)	
High school	11 (5.6)	6 (6.2)	5 (5.1)	
University or higher	29 (14.9)	16 (16.7)	13 (13.1)	
Don’t know	101 (51.8)	64 (66.7)	37 (37.4)	

*Values are number (%) for categorical variables and mean (standard deviation) for continuous variables*

^*^ *Chi-square test or Fisher’s exact test as appropriate was used for continuous variables, and Student’s t-test for continuous variables*

#### ART, immunological and virological statuses, and healthcare coverage

Table 2 shows that 82.4% were on first line ART. On average, the participants had received treatment and care services from an ART clinic for 9.5 (SD = 3.3) years, and received ART for 8.4 (SD = 3.3) years. The average travel expenditure to the ART clinic was $3.4 (SD = 3.9). In addition, 95.6% were adherent to ART based on self-reported Visual Adherence Scale.

**Table 2:** ART, immunological and virological statuses and healthcare coverage of adolescents living with HIV from 11 ART clinics in Cambodia

	Total (*n* = 328)	Males (*n* = 169)	Females (*n* = 159)	
	*n* (%)	*n* (%)	*n* (%)	*P*-value^*^
*Antiretroviral therapy (ART)*				
Mean travel time to ART clinic (min)	48.2 ± 43.4	46.1 ± 41.2	55.5 ± 45.5	0.36
Type of ART regimen				0.91
First line	183 (82.4)	140 (82.8)	131 (82.4)	
Second line	36 (16.2)	29 (17.2)	27 (17.6)	
Time since ART started (in years)	8.2 ± 3.3	8.3 ± 3.4	7.8 ± 3.3	0.20
Mean cost to travel to ART clinic (USD)	3.4 ± 3.9	3.5 ± 3.9	3.3 ± 4.0	0.61
Visual Adherence Scale (%)	95.4 ± 9.6	95.3 ± 9.6	95.4 ± 9.7	0.91
*Immunological and virological status*				
Initial CD4 count (mean cells/mm^3^)	632 ± 1101	777 ± 1460	632 ± 460	0.35
Latest CD4 count (mean cells/mm^3^)	672 ± 284	683 ± 314	660 ± 250	0.44
First viral load count (copies)	34,375 ± 13,958	37,868 ± 13,205	30,662 ± 14,748	0.64
Latest viral load count (copies)	9268 ± 6407	4103 ± 1573	14,759 ± 9041	0.13
Viral suppression	252 (76.8)	131 (77.5)	121 (76.1)	0.76
*Healthcare access and coverage*				
Ability to cover health expenses				0.11
No	181 (55.2)	86 (50.9)	95 (59.7)	
Yes	147 (44.8)	83 (49.3)	64 (40.3)	
Family has an ID poor card				0.04
No	190 (57.9)	88 (52.1)	102 (64.2)	
Yes	112 (34.2)	63 (37.3)	49 (30.8)	
Don’t know	26 (7.9)	18 (10.7)	8 (5.0)	
Access to the health equity fund				< 0.001
No	185 (56.4)	109 (64.5)	76 (47.8)	
Yes	126 (38.4)	45 (35.7)	81 (50.9)	
Don’t know	17 (5.2)	15 (8.9)	2 (1.3)	
Covered by a health insurance scheme				0.02
No	289 (88.1)	148 (87.6)	141 (88.7)	
Yes	6 (1.8)	0 (0.0)	6 (3.8)	
Don’t know	33 (10.1)	21 (12.4)	12 (7.5)	

*Values are number (%) for categorical variables and mean (standard deviation) for continuous variables*

^*^ *Chi-square test or Fisher’s exact test as appropriate was used for continuous variables, and Student’s t-test for continuous variables*

The mean CD4 count in the first test was 632 (SD = 506) cells/mm^3^, and in the most recent test was 672 (SD = 295) cells/mm^3^. Mean viral load count in the initial test was 44,691 (SD = 161,304) copies/mL, and in the most recent test was 7686 (SD = 48,682) copies/mL. The proportion of participants with viral suppression (viral load count < 1000 copies/mL) was 76.8%. No significant difference was found in ART and immunological and virological statuses among male and female participants.

Less than half (44.8%) of the participants reported that their family was able to cover their health expenses without support. About one-third (34.2%) had an ID poor card; 38.4% had access to the health equity fund; and only 1.8% had their treatment covered by a health insurance scheme. Significant gender differences was found in the proportion of family having an ID poor card (*p* = 0.04), access to health equity fund (*p* ≤ 0.001), and having their treatment covered by a health insurance scheme (*p* = 0.02).

#### Awareness and disclosure of HIV status

As shown in Tables 3, 94.5% were aware that they were living with HIV. About half (49.1%) of the participants had disclosed their HIV status to someone in their social circles, mostly to siblings or friends. Half (50.7%) had never disclosed their HIV status to anyone. In addition, 82.6% responded that they had acquired the HIV infection from their mothers, while 17.4% did not know the source of their HIV infection. When asked about modes of HIV transmission, 96.7% knew at least one mode of transmission. Significant gender differences were found in knowledge of HIV transmission modes (*p* = 0.04 for mother to child transmission, and *p* = 0.001 for blood transfusion) and disclosure status (*p* = 0.03).

**Table 3:** Awareness and disclosure of HIV status among adolescents living with HIV from 11 ART clinics in Cambodia

	Total (*n* = 328)	Males (*n* = 169)	Females (*n* = 159)	
	*n* (%)	*n* (%)	*n* (%)	*P*-value^*^
*General understanding about HIV and AIDS*				
What is your disease?				0.19
HIV/AIDS	310 (94.5)	157 (92.9)	153 (96.2)	
Don’t know	18 (5.5)	12 (7.1)	6 (3.8)	
Do you know how is this disease transmitted?				
Don’t know	8 (2.6)	5 (3.2)	3 (2.0)	0.5
Mother to child	207 (67.4)	114 (72.0)	95 (62.1)	0.04
Unprotected sex	236 (76.1)	119 (75.8)	117 (76.5)	0.89
Sharing needles	178 (57.4)	90 (57.3)	88 (57.5)	0.97
Blood transfusion	191 (61.6)	82 (52.2)	109 (71.2)	0.001
Other	65 (21.0)	35 (22.3)	30 (19.6)	0.56
*Awareness about self and disclosure*				
Do you know how you have been infected?				0.97
From mother	256 (82.6)	189 (82.2)	127 (83.0)	
Don’t know	54 (17.4)	28 (17.8)	26 (17.0)	
Do you know what kind of medicine you have received?				0.16
Pre-ART	6 (1.9)	1 (0.6)	5 (3.3)	
ART	268 (86.5)	141 (89.2)	127 (83.6)	
Don’t know	36 (11.6)	16 (10.1)	20 (13.2)	
Have you ever disclosed your HIV status to anyone?				0.03
None	167 (54.0)	77 (48.7)	90 (59.6)	
Siblings	72 (23.3)	47 (29.7)	25 (16.6)	
School teachers	6 (1.9)	3 (1.9)	3 (2.0)	
Friends	44 (14.2)	18 (11.4)	26 (17.2)	
Other (please specify)	20 (6.5)	13 (8.2)	7 (4.6)	

*Values are number (%) for categorical variables and mean (standard deviation) for continuous variables*

^*^ *Chi-square test or Fisher’s exact test as appropriate was used for continuous variables, and Student’s t-test for continuous variables*

#### Sexual behaviors and substance use

As shown in Table 4, about a fifth (20.7%) of participants reported having had a boy or girlfriend, but only 2.1% reported having had sexual intercourse in the past 12 months. None of those who had sexual experience reported using a condom in their last intercourse. Very few reported having used tobacco (1.8%), or any kind of illicit drugs (0.9%), but almost a fifth had a history of alcohol use. Apart from experience in sexual intercourse (*p* = 0.04), there was no significant gender difference in other variables.

**Table 4:** Sexual behaviors and substance use among adolescents living with HIV from 11 ART clinics in Cambodia

	Total (*n* = 328)	Males (*n* = 169)	Females (*n* = 159)	
	*n* (%)	*n* (%)	*n* (%)	*P*-value^*^
Sexual behaviors				
Had a boy (girl) friend in the past 12 months				0.41
No	260 (79.3)	137 (81.1)	123 (77.4)	
Yes	68 (20.7)	32 (18.9)	36 (22.6)	
Had sexual intercourse in the past 12 months				0.04
No	321 (97.9)	168 (99.4)	153 (96.2)	
Yes	7 (2.1)	1 (0.6)	6 (3.8)	
Condom use at last sexual intercourse				NA
No	7 (100)	1 (100)	6 (100)	
Yes	0 (0.0)	0 (0.0)	0 (0.0)	
Substance use				
Ever smoked tobacco				0.12
No	322 (98.2)	164 (97.0)	158 (99.4)	
Yes	6 (1.8)	5 (3.0)	1 (0.6)	
Ever used alcohol				0.07
No	267 (81.4)	144 (85.2)	123 (77.4)	
Yes	61 (18.6)	25 (14.8)	36 (22.6)	
Ever used any kind of illicit drugs				0.07
No	325 (99.1)	169 (100)	156 (98.1)	
Yes	3 (0.9)	0 (0.0)	3 (1.9)	

*Values are number (%) for categorical variables and mean (standard deviation) for continuous variables*

^*^ *Chi-square test or Fisher’s exact test as appropriate was used for continuous variables and Student’s t-test for continuous variables*

#### HIV knowledge

Table 5 shows that level of HIV knowledge among adolescents in this study was generally high, although there were still a good number of adolescents who did not agree or know HIV is a virus that attacks a person’s immune system (19.8%), the function of CD4 cells of fighting infection (38.1%), or the amount of HIV in a person’s blood is called viral load (51.2%). In addition, 49.8% believed that there is a cure for AIDS. However, the majority of the participants were of the view that you cannot tell a person has HIV by looking at him or her (82%), and that it is not okay for people living with HIV to miss their medicines (83.5%). A significant number disagreed with the statement that “If people living with HIV take medicines, they don’t have to use condoms” (58.8%) and “If sexual partners are both living with HIV, they don’t have to use a condom” (47.3%), among other findings. Significant gender differences were found in HIV knowledge, especially in understanding about CD4 (*p* = 0.03), viral load (*p* = 0.004), condom use (*p* = 0.005), and missing ARV dose (*p* = 0.02).

**Table 5:** HIV knowledge among adolescents living with HIV from 11 ART clinics in Cambodia

HIV knowledge	Total (*n* = 328)	Males (*n* = 169)	Females (*n* = 159)	
	*n* (%)	*n* (%)	*n* (%)	*P*-value^*^
HIV is a virus that attacks a person’s immune system				0.45
True (correct)	263 (80.2)	140 (82.8)	123 (77.4)	
False	44 (13.4)	20 (11.8)	24 (15.1)	
Don’t know	21 (6.4)	9 (5.3)	12 (7.5)	
The cells of the immune system that fight infection are called CD4 cells				0.03
True (correct)	203 (61.9)	116 (68.6)	87 (54.7)	
False	22 (6.7)	8 (4.7)	14 (8.8)	
Don’t know	103 (31.4)	45 (26.6)	58 (36.5)	
The amount of HIV in a person’s blood is called viral load				0.004
True (correct)	160 (48.8)	97 (57.4)	63 (39.6)	
False	26 (7.9)	13 (7.7)	13 (8.2)	
Don’t know	142 (43.3)	59 (34.9)	83 (52.2)	
There is a cure for AIDS				0.38
True (correct)	138 (42.1)	67 (39.6)	71 (44.7)	
False	165 (50.3)	93 (53.8)	74 (46.5)	
Don’t know	25 (7.6)	11 (6.5)	14 (8.8)	
You can tell a person has HIV by looking at him or her				0.09
True (incorrect)	46 (14.0)	29 (17.2)	17 (10.7)	
False	269 (82.0)	136 (80.5)	133 (83.6)	
Don’t know	13 (4.0)	4 (2.4)	9 (5.7)	
A person with HIV will stay healthy if their CD4 cells are high and their viral load is low				0.99
True (correct)	284 (86.6)	146 (86.4)	138 (86.8)	
False	23 (7.0)	12 (7.1)	11 (6.9)	
Don’t know	21 (6.4)	11 (6.5)	10 (6.3)	
Even though a person with HIV may feel healthy, the virus can still be damaging his or her immune system				0.86
True (correct)	248 (75.6)	127 (75.1)	121 (76.1)	
False	65 (19.8)	35 (20.7)	30 (18.9)	
Don’t know	15 (4.6)	7 (4.1)	8 (5.0)	
If sexual partners are both living with HIV, they don’t have to use condoms				0.07
True (incorrect)		61 (36.1)	64 (40.3)	
False		89 (52.7)	66 (41.5)	
Don’t know		19 (11.2)	29 (18.2)	
If people living with HIV take medicine, they don’t have to use condoms				0.005
True (incorrect)	86 (26.2)	41 (24.3)	45 (28.3)	
False	193 (58.8)	112 (66.3)	81 (50.9)	
Don’t know	49 (14.9)	16 (9.5)	33 (20.8)	
It is okay for people living with HIV to miss their medicines				0.02
True (incorrect)	43 (13.1)	14 (8.3)	29 (18.2)	
False	277 (83.5)	148 (87.6)	126 (79.2)	
Don’t know	11 (3.4)	7 (4.1)	4 (2.5)	

*Values are number (%) for categorical variables and mean (standard deviation) for continuous variables*

^*^ *Chi-square test or Fisher’s exact test as appropriate was used for continuous variables and Student’s t-test for continuous variables*

## Discussion

This paper reports important characteristics of adolescents living with HIV aged 15–17 years old, who remained in pediatric care and in preparation for transition into adult HIV care. Several findings which are central to WHO’s definition of healthcare transition readiness are notable. Some gender differences in these characteristics were also identified.

In this study, the majority of adolescents were still on first line treatment, and most reported a high level of adherence to ART on Visual Analogue Scale (95.4%). Adherence is a significant determinant of treatment success [7], and an indicator of healthcare transition readiness as part of self-health management [26, 42]. Our finding of high ART adherence among the sample is a departure from frequent reports of much lower rates of adherence among adolescents living with HIV studied elsewhere [7, 24] and low level of adherence among children and adolescents living with HIV in general [43]. In other studies, treatment fatigue [8], side effects, anticipated stigma, fear of HIV status disclosure, psychological factors, client-provider relationship [24, 44, 45], and inconsistent daily routine [46] contributed to low adherence among older adolescents. A recent review also suggested that only 70% of adolescents and young adults living with HIV in Africa and Asia receiving ART were adherent to it [47].

A possible explanation to the high level of adherence reported in our study may be related to the widely available free HIV services including ART and social support (e.g. 34.2% and 38.2% having a poor ID card and health equity fund, respectively), despite the fact that many had lost at least one of their parents and were from a poor family background. Availability of social and financial protection can affect treatment adherence and preparation to transition [24, 46]. Likewise, inability to cover medical prescription, partial insurance coverage and other economic barriers, and lack of access to insurance, among others, are threats to successful healthcare transition, even among adolescents who have ‘developmental readiness’ for transition, according to other research studies [21, 25, 48].

However, we noted that despite the high level of adherence reported, the average viral load was high (7686 copies/mL). One of the possible explanations for this is the time lag between the viral load and the adherence measurements. While the adherence was assessed over the last three days, medical records were used to determine the last viral load measurements, which take place six monthly for new and unstable patients and annually for stable patients. It is also possible that the ART adherence levels reported in our study were overestimated due to social desirability bias and the subjective nature of the visual adherence scale, which may have led adolescents to report higher than actual adherence. An assessment of viral load levels using more objective methods such as plasma drug levels will be required in future studies to ascertain levels of adherence. Although other studies have reported loss to follow up, transfer out, and mortality outcomes during and following transition [22, 49–51], these data were not collected in our study and will need to be tracked.

Our study suggests that, although most adolescents were well informed of its nature and how they acquired HIV, there were still significant gaps in the awareness of HIV risk factors and some of the specifics related to viral and immunological responses to HIV. The lack of appropriate information has been reported in other studies of adolescents transitioning into adult care [52]. While lack of HIV knowledge is not unique among adolescents living with HIV [52, 53], a good understanding of HIV and its natural history is a critical part of treatment literacy and self-management skills, and therefore gains prominence in relation to older adolescents who are about to take responsibility over their own health. As a consequence, and in line with WHO’s understanding of transition readiness [26], additional focus on HIV treatment literacy will be required as part of the transition preparedness process.

Half of the adolescents had not disclosed their HIV status to anyone, while 23.3% disclosed it to their sibling and 14.2% to their friends, and 5.5% of the adolescents did not know their HIV status. Adolescents require information and ongoing support to determine how, when, and whom to disclose, enabling them to make informed decisions, balancing the risks and benefits of disclosure [54]. Studies of adolescents in transition clinics have shown that adolescents face challenges with disclosure [11]. Among both transitioning and other adolescents in general, disclosure can lead to negative consequences of rejection and stigmatization, even from the loved ones [12, 13, 37, 55] and in other cases, it can open opportunities for support from family members and intimate partners [13], participation in peer-support groups [10], and management of sexual risks [56]. It is widely accepted that besides access to ART, adolescent patients need attention and care to their ‘physical and psychological needs’ from their immediate families and broader social groups and adolescent-centered approaches to treatment [55], and this is especially the case for those who have lost their parent(s) – 60.9% of the adolescents in this study lost their mothers and 60.4% their fathers. Because of these tensions, efforts to assist adolescents to make informed decisions on HIV status disclosure should form a central part of regular communication with adolescents who are getting ready for transition in Cambodia, as argued by other researchers elsewhere [18].

In particular, there is a need to ensure that these adolescents have the skills to disclose should they want to [54]. In Cambodia, case managers, who manage transition for adolescents, have a significant role to play as a source of advice, information, and communication skills regarding disclosure by adolescents to their intimate partners and other peers. This will require concerted efforts to involve care givers to provide age-appropriate disclosure to the few adolescents who were not aware of their status, ensuring that those who are taking care of them, whether biological parents or not, are involved and understand the need for confidentiality. The need to strengthen adolescents’ disclosure skills, especially as part of transition, is not unique to Cambodia, having been identified elsewhere [46]. In addition, WHO recommends that age appropriate disclosure should accompany self-management of HIV [26]. Although adolescents in this study are generally not yet sexually active, but given their transition into adulthood, other important health knowledge and skills including sexual and reproductive health such as use of condoms and other birth control methods, use of illicit substances, and healthy lifestyles should be included.

Few if any studies have explored gender differences among transitioning adolescents. However, our data suggested gender differences in some of the characteristics of adolescents we studied. Although very few female adolescents in the study were sexually active at their age, which warrants cautious interpretation, more females than males (or 6 female over 1 male) had had early sexual debut in keeping with universal observations of earlier sexual maturity among female adolescents living with HIV [57]. Early sexual debut (sex before 15 years of age) is more common among adolescent girls than boys in low- and middle-income countries [6].

However, when we consider that female adolescents living with HIV also seemed to have slightly lower levels of general knowledge about HIV (Table 5), it raises concerns regarding potential differences in sexual risks, for instance of acquiring other HIV strains, or of transmitting the virus unknowingly. Understanding sexual risks is a critical part of ensuring that adolescents can independently manage their health. This suggests that healthcare transition interventions in the study context should address issues of sexual risks, sexuality, and sexual negotiation among this population as they mature. There is general consensus that skills to empower adolescents to negotiate sexual relationships are required as adolescents living with HIV physically and psychologically develop [18, 58].

In addition, we emphasize that within these gender categories, differences between older and younger adolescents may also exist as documented elsewhere [59]. Age alone may not always be a good predictor of healthcare transition readiness, but should be considered alongside individual adolescent’s evolving needs as part of a gradual process in which they prepare for transition [24, 50, 60]. As such the adolescents aged 15–17 preparing for transition in Cambodia and elsewhere should not be viewed as a homogenous group, but rather a dynamic, heterogeneous group with differing gender and sexual preferences, stigma, adherence, and other social protection and financial concerns within a diverse social context. To provide individualized support for transition, pediatric and adult clinics will need to ensure that these unique individual, social, and structural characteristics are taken into account as argued by other researchers in Cambodia and other contexts [17, 32, 46, 48, 61], ensuring that context specific differences are taken into account. As other researchers have argued, this requires different care and treatment arrangements and options; transition planning, process, procedure, and execution; inter-clinic communication and data sharing as well as coordination and communication with and among adolescents, their parents and caretakers, and multidisciplinary health and social teams [24, 62, 63].

Finally, and while our study did not look at wider clinical processes in depth, findings from other contexts suggest that inter-clinic communication, clinical administration processes, differences in care cultures, and personnel deployments are all useful determinants of successful transition [64], which should be taken into account in the Cambodian context.

### Study limitations

Some of the characteristics reported relied on self-reported measures, which could be affected by social desirability and recall biases. Our study was conducted among adolescents living with HIV who were receiving health services in public or NGO clinics; hence, our findings may not be generalized to all adolescents living with HIV in the country. In addition, given the high cost in collecting data from low volume clinics, adolescents who accessed care from clinics providing care to less than 10 adolescents were excluded, which may bias results, given that volume of patients is a key determinant of provider competency [65] and patient outcomes [66]. Our study was cross sectional in nature and does not report the temporal variations of the variables. It is possible that some of these measures could change over time.

## Conclusions

Understanding characteristics and circumstances of adolescents living with HIV is critical in designing adolescent-friendly services, including healthcare transition interventions. This study provides a snapshot of immunological, virological, adherence, and disclosure outcomes among adolescents preparing for transition into adult clinics. Several findings are consistent with the literature, including low likelihood of disclosure outside of family circles, experience of HIV stigma, and sub-optimal condom use and poor knowledge of HIV. This study adds to the growing literature on immunological, virological, HIV knowledge disclosure, and adherence characteristics of adolescents preparing for transition from pediatric to adult services. There is also added value in tracking these and other characteristics during and following transition to understand the effectiveness of transition program in Cambodia.

Based on WHO’s definition of transition preparedness [26], our recommendations fall into three categories. First is to strengthen adolescents’ ability to demonstrate basic healthcare awareness through educating them on HIV. Second is to enable adolescents to understand their disease by specifically enabling those who are not disclosed to know their HIV status. And third is to support them to develop their health management skills by involving them in tracking their own immunological, virological, and other clinical outcomes as well as non-clinical outcomes such as adherence and self-management skills.

## Abbreviations

ART: Antiretroviral therapy
HIV: Human immunodeficiency virus
NECHR: National Ethics Committee for Health Research
SD: Standard deviation
STD: Sexually transmitted diseases
WHO: World Health Organization
